# Efficacy of a Modular App-Based Pelvic Floor Muscle Training Program for Postoperative Continence Recovery After Radical Prostatectomy: A Multi-Center Randomized Controlled Trial (PELVINTENSE Study)

**DOI:** 10.3390/cancers18091333

**Published:** 2026-04-22

**Authors:** Bara Barakat, Mustapha Addali, Sameh Hijazi, Saed Alqaddi, Christian Rehme, Boris Hadaschik, Sabine D. Brookman-May

**Affiliations:** 1 Department of Urology, Hospital Kassel, 34125 Kassel, Germany; bara.barakat@gnh.net; 2 Department of Urology and Pediatric Urology, Hospital Viersen, 41747 Viersen, Germany; 3 Department of Urology, Hospital Siegen, 57076 Siegen, Germany; m.addali@klinikum-siegen.de; 4 Department of Urology, Hospital Ibbenbüren, 49477 Ibbenbüren, Germany; s.hijazi@mathias-spital.de (S.H.); s.alqaddi@mathias-stiftung.de (S.A.); 5 Department of Urology, University Cologne, 50937 Cologne, Germany; 6 Department of Urology, University Hospital Essen, 45147 Essen, Germany; christian.rehme@uk-essen.de (C.R.); boris.hadaschik@uk-essen.de (B.H.); 7 Department of Urology, Ludwig Maximilian University (LMU), 81377 Munich, Germany; 8 Aura Biosciences, Inc., Boston, MA 02135, USA

**Keywords:** app-based interventions, digital health technology, patient adherence, prehabilitation, prostate cancer, urinary incontinence, quality of life

## Abstract

**Background/Objectives:** Stress urinary incontinence (SUI) remains a common and functionally relevant complication after radical prostatectomy (RP) and substantially impairs quality of life (QoL). Although pelvic floor muscle training (PFMT) is guideline-recommended, its real-world effectiveness is often limited by accessibility, standardization, and adherence. Digital health interventions may improve adherence to PFMT, potentially influencing continence recovery. We conducted a multicenter randomized controlled trial to evaluate whether a structured, modular app-based PFMT program improves early continence recovery compared with conventional physiotherapist-guided training. **Methods:** Between September 2022 and September 2024, 62 preoperatively continent men undergoing radical prostatectomy were enrolled in this multicenter randomized controlled trial (Pelvintense). Both groups received perioperative PFMT: Patients were randomized 1:1 to either a modular app-based PFMT program (intervention group) or a standard physiotherapist-guided PFMT (control group). Both app-based PFMT and standard physiotherapist-guided PFMT started three weeks before surgery and continued for 90 days postoperatively. The primary endpoint was continence at 90 days, defined as ICIQ-SF Q1 = 0 (absence of involuntary SUI). Secondary endpoints included continence sub-scores, QoL, erectile function, adherence, and decision regret. Analyses were performed using a modified intention-to-treat approach applying logistic regression and non-parametric tests with sensitivity analyses. **Results:** A total of 62 patients were included in the study and randomized, with 31 allocated to the app-based PFMT arm and 31 to the standard physiotherapist-guided arm. Three patients in the control arm withdrew consent for data usage after randomization, resulting in a modified intention-to-treat population of 59 patients. At 90 days, continence rates were higher in the app-based group compared with the control group (74.2% versus 21.4%; *p* < 0.001), corresponding to an absolute risk reduction of 52.8% and a number needed to treat of two. In multivariable analysis, participation in the app-based program was independently associated with higher odds of continence recovery (odds ratio 13.80, 95%-confidence interval 3.22–59.12; *p* < 0.001). Continence at 30 days and continence-related QoL favored the intervention, whereas no significant differences were observed in erectile function at 90 days. Adherence to the PFMT was higher in the intervention group. Sensitivity analyses confirmed the robustness of the primary outcome. **Conclusions:** In this randomized controlled trial, a modular app-based PFMT program was associated with early continence recovery after prostatectomy compared with the standard-of-care physiotherapist-guided PFMT. Improved adherence, modular progression of exercises, and a more structured training delivery may have contributed to the effect. App-based PFMT might represent a scalable strategy to implement guideline-recommended supportive care. These findings warrant confirmation in studies with a longer follow-up.

## Simple Summary

After radical prostatectomy, many men experience stress urinary incontinence, which can strongly affect daily activities and quality of life. Pelvic floor muscle training is recommended to improve continence, but access to physiotherapy and long-term adherence are often limited. Digital health tools may help deliver structured training more consistently. In this multicenter randomized study, we compared an app-based perioperative pelvic floor muscle training program with standard physiotherapist-guided training. Men scheduled for radical prostatectomy started training three weeks before surgery and continued until three months afterward. The main outcome was whether patients were completely continent 90 days after surgery. We also assessed continence severity, quality of life, treatment satisfaction, and adherence. Patients using the app recovered continence faster and more often than those receiving standard care. At three months, about three-quarters of app users were continent compared with about one-fifth of patients in the control group. This benefit remained stable in additional analyses that tested conservative assumptions. App users also reported better continence-related quality of life and showed higher adherence before surgery, while erectile function did not differ between groups. These findings indicate that app-based pelvic floor muscle training is associated with significantly improved early recovery after prostate surgery and supports scalable care delivery.

## 1. Introduction

Radical prostatectomy (RP) remains the most frequently performed curative treatment for men with localized prostate cancer (PCa) [1,2]. Despite substantial refinements in surgical technique, perioperative management, and nerve-sparing strategies, stress urinary incontinence (SUI) continues to represent one of the most prevalent and burdensome postoperative sequelae. SUI has a profound impact on quality of life (QoL), patient satisfaction, and perceived success of treatment, often outweighing oncological outcomes in patient-reported priorities [3,4,5,6,7,8]. Although continence recovery may occur gradually over time, complete restoration can take up to 24 months and remains incomplete in a considerable proportion of patients, even in contemporary surgical series and structured perioperative care pathways [7,8,9,10,11]. Beyond its immediate functional consequences, post-prostatectomy incontinence should be considered within a broader framework of chronic functional impairment, with potential implications for long-term disability, healthcare utilization, and the need for sustained multidisciplinary care. Recent data emphasize that patients with functional limitations frequently require complex healthcare pathways integrating medical treatment, rehabilitation, and social support systems [12].

Pelvic floor muscle training (PFMT) is recommended as the first-line conservative intervention for post-prostatectomy SUI by both the European Association of Urology (EAU) and the American Urological Association (AUA) [8]. Physiotherapist-guided PFMT has been shown to improve continence outcomes; however, in routine clinical practice, training programs are frequently initiated only after surgery and vary substantially in structure, intensity, and adherence, as reported in prior clinical studies and systematic reviews. This postoperative focus fails to address preoperative pelvic floor muscle conditioning, which is increasingly recognized as a relevant determinant of early functional recovery. In addition, adherence to physiotherapist-guided PFMT in routine practice is often inconsistent, further limiting its effectiveness outside controlled study settings. Over the past decade, accumulating evidence has supported a perioperative or prehabilitation-based approach that integrates pre- and postoperative PFMT to accelerate continence recovery following RP [12,13,14,15,16,17]. Meta-analyses have consistently demonstrated superior early continence outcomes with combined perioperative training compared with postoperative PFMT alone, particularly with respect to time-to-continence recovery [18,19,20]. Nevertheless, standardized perioperative PFMT protocols have rarely been evaluated within adequately powered, high-quality randomized controlled trials.

In parallel, the expanding adoption of mobile health technologies has created new opportunities to deliver rehabilitation interventions in a scalable, patient-centered, and resource-efficient manner. App-based PFMT programs have been associated with improved adherence, sustained engagement, and favorable QoL outcomes, while reducing logistical barriers inherent to traditional in-person physiotherapy [21]. Digital platforms further offer the potential to standardize training content, reinforce correct exercise execution through audiovisual guidance, and support continuous perioperative engagement, which are critical determinants of behavioral interventions.

Beyond rule-based digital interventions, artificial intelligence (AI) is increasingly shaping the next generation of digital health solutions in oncology. AI-driven systems have demonstrated the potential to personalize treatment pathways, predict functional recovery, and optimize patient engagement by integrating longitudinal behavioral and clinical data. In the context of perioperative care, such approaches may enable dynamic adaptation of rehabilitation protocols, identification of patients at risk for delayed recovery, and data-driven optimization of supportive care strategies. Embedding structured digital interventions within an AI-enabled framework, therefore, represents a promising avenue toward precision rehabilitation in urologic oncology.

However, the current evidence base remains limited, with most available studies being small, single-center investigations or lacking randomized controlled designs. In particular, there is a paucity of multicenter randomized trials evaluating fully digital, structured PFMT interventions in the perioperative setting. The present multicenter randomized controlled study was therefore designed to address this gap by evaluating the efficacy of Pelvintense, a standardized, modular app-based audiovisual perioperative PFMT program in men undergoing RP. We hypothesized that digital delivery of a structured perioperative PFMT regimen would result in earlier recovery of urinary continence, defined by patient-reported absence of involuntary urine leakage and improved postoperative QoL when compared with standard physiotherapist-guided perioperative training.

## 2. Materials and Methods

### 2.1. Study Objectives and Design

The Pelvintense study is a prospective, multicenter, two-arm randomized controlled trial designed to evaluate whether a modular, app-based perioperative PFMT program (Pelvintense; Vitruvo eHealth GmbH, Regensburg, Germany) improves the early recovery of urinary continence after RP compared with conventional physiotherapist-guided training. The primary objective was to assess the recovery of SUI at 90 days following the surgery. Secondary objectives included the evaluation of health-related QoL, decision regret, adherence to the assigned training program, and early erectile function recovery. Exploratory objectives included patient engagement and the feasibility of a scalable digital perioperative rehabilitation model.

The digital intervention applied in this study, i.e., the Pelvintense app, further allows structured longitudinal data collection on patient adherence and functional outcomes, providing a foundation for future AI-driven modeling of recovery trajectories and personalized rehabilitation strategies.

The trial was conducted across three German urological centers (Siegen, Viersen, and Ibbenbüren), prospectively registered in the German Clinical Trials Register (DRKS00025467), and performed in accordance with the Declaration of Helsinki. Ethical approval was obtained from the Ethics Committee of the University of Duisburg-Essen (protocol code 20-9702-BO; date of approval: 17 June 2021) and from the local ethics committees of all participating centers. Patient enrollment took place between September 2022 and September 2024. All participants provided written informed consent prior to their inclusion in the study.

### 2.2. Participants and Eligibility Criteria

Eligible participants were preoperatively continent men with localized prostate cancer (PCa) scheduled to undergo RP. Preoperative continence was defined as the absence of involuntary urine leakage. Exclusion criteria included cognitive impairment, any degree of pre-existing urinary incontinence, prior pelvic radiation therapy, and medical or functional conditions expected to preclude adherence to the assigned training protocol.

RP was performed by experienced surgeons using open, laparoscopic, or robotic-assisted approaches according to institutional standards. Nerve-sparing techniques were applied when clinically appropriate. Surgical technique and the distribution of unilateral nerve-sparing, bilateral nerve-sparing, and non-nerve-sparing procedures were comparable between the two study arms. Baseline demographic, clinical, and surgical characteristics, including surgical approach, nerve-sparing status, surgeon experience, and postoperative complication rates, were prospectively recorded (Table 1).

### 2.3. Randomization and Study Procedures

Participants were randomized in a 1:1 ratio to the digital intervention arm or the standard physiotherapist-guided training arm using a computer-generated allocation sequence with a predefined block size of four and a 1:1 allocation ratio. Allocation concealment was ensured through centralized assignment prior to surgery. Randomization was performed four weeks prior to surgery. Due to the behavioral nature of the intervention, the blinding of participants and treating clinicians was not feasible. However, the outcome assessment was based on standardized patient-reported measures to minimize assessment bias. Both training programs commenced three weeks before RP and continued until 90 days postoperatively. Standard postoperative inpatient rehabilitation was offered to participants in both study arms according to institutional practice.

### 2.4. App-Based PFMT

In the control group, PFMT was performed according to standard physiotherapist-guided protocols, typically consisting of in-person instruction and home-based exercises without structured digital support.

**Figure 1:**
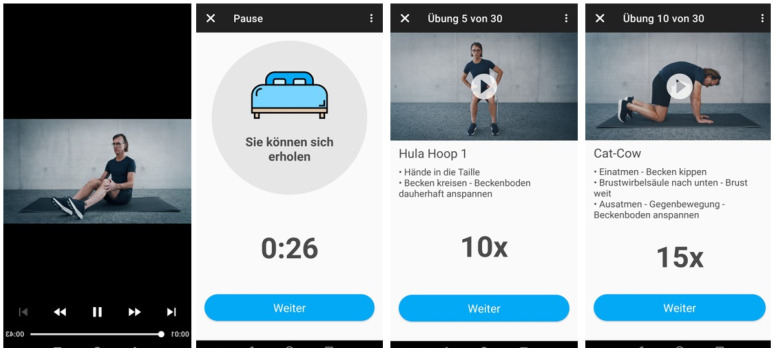
Representative screenshots from the Pelvintense app illustrating key components of the modular training program. The app delivers audiovisual guidance for each exercise, integrates structured rest periods, and provides real-time instruction on movement execution, repetition counts, and pelvic floor activation. The interactive format standardizes training techniques, fosters user engagement, and supports adherence throughout the perioperative prehabilitation and rehabilitation phase. Translation of German guidance (images from left to right and translations from top to bottom): Image 2: Pause—Take time to recover—Proceed. Image 3: Exercise 5 of 30—Hula Hoop 1: Hands on the waist; circle the pelvis; keep the pelvic floor continuously engaged—Proceed. Image 4: Exercise 10 of 30—Cat–Cow: Inhale—tilt the pelvis, drop the thoracic spine downward, open the chest; exhale—reverse the movement and engage the pelvic floor—Proceed.

Intervention Arm (app-based perioperative PFMT; Pelvintense app): Participants allocated to the intervention arm received training via the Pelvintense application (Vitruvo eHealth GmbH, Regensburg, Germany), a certified Class I medical device under the European Medical Device Regulation. The application delivers an audiovisual perioperative PFMT program consisting of modular training units with progressive levels of difficulty. The app-based PFMT program consisted of structured, progressive training modules including guided pelvic floor contractions, relaxation, and coordination exercises. The program incorporates interactive animations, structured exercise progression, and automated adherence tracking (Figure 1). Training intensity and progression were adapted individually, with progressive intensity adjustments based on user performance and adherence. To progress to the next module and intensity level, participants must have trained for at least two weeks on the previous level within modules 1–4 and completed a minimum of 15 individual training sessions; the final module, module 5, requires training at varying intensity levels for at least three weeks to complete the training program. Participants received standardized verbal and written instructions at the baseline, and adherence was continuously monitored using in-app logging functions.

Control Arm (physiotherapist-guided perioperative PFMT): Participants in the control arm received standard perioperative PFMT under the supervision of a physiotherapist. The intervention consisted of in-person instruction sessions combined with written guidance for home-based exercises. The temporal structure of training mirrored that of the intervention arm, with initiation three weeks before RP, resumption after urinary catheter removal, and continuation for 90 days following surgery. All participants received standardized instruction in correct pelvic floor muscle activation and were encouraged to perform daily exercises throughout the study period.

### 2.5. Outcome Measurements and Endpoints

The primary endpoint was a recovery of SUI at 90 days after RP, defined as the absence of involuntary urinary leakage. Continence was operationalized using item Q1 of the International Consultation on Incontinence Questionnaire in short form (ICIQ-SF), with a binary definition of continence (ICIQ-SF Q1 = 0) versus persistent incontinence (ICIQ-SF Q1 ≥ 1). This definition was selected to represent a stringent and clinically meaningful endpoint corresponding to a complete absence of patient-reported urinary leakage and to ensure a standardized and reproducible assessment of continence recovery across study participants.

Secondary endpoints included a continence status at week 1 and at 30 days postoperatively, as well as early erectile function assessed using the International Index of Erectile Function in short form (IIEF-5). Exploratory endpoints comprised continence-related sub-scores of the ICIQ-SF (items Q1–Q3), QoL assessed by IPSS item Q8, decision regret measured using the decision regret scale at day 90, and adherence metrics reflecting engagement with the assigned training program [22,23,24,25]. In scope with its objectives, the study was not powered to detect differences in safety outcomes.

### 2.6. Statistical Analyses

Sample size estimation was based on an expected continence rate at day 90 of 75% in the intervention arm and 40% in the control arm [26]. Assuming a one-sided alpha level of 0.05 and a power of 80% (β = 0.20), a minimum of 24 participants per group was required. To account for an anticipated attrition rate of up to 25%, the target sample size was increased to 62 participants, corresponding to 31 patients per study arm.

Baseline characteristics were summarized using descriptive statistics. Continuous variables are reported as medians with interquartile ranges (IQR), and categorical variables as absolute frequencies and percentages. Between-group comparisons were performed using the Mann–Whitney U test for non-normally distributed continuous variables and the chi-square test for categorical variables.

The primary endpoint was predefined as continence at 3 months. The primary endpoint was analyzed using logistic regression to estimate group differences in continence status at day 90. Odds ratios (OR) with 95% confidence intervals (CI) were reported. Univariable binary logistic regression analyses were performed to identify factors associated with continence recovery at 90 days, defined as ICIQ-SF Q1 = 0. To account for the underlying data structure and avoid a priori exclusion of potentially relevant covariates, all variables demonstrating an association with the primary endpoint at a threshold of *p* < 0.20 in univariable analyses were entered into the multivariable logistic regression model. Model fit was assessed using the Hosmer–Lemeshow goodness-of-fit test and Nagelkerke’s R^2^. In addition, potential multicollinearity between covariates was assessed, and the number of variables included in the multivariable model was restricted relative to the number of outcome events to reduce the risk of model overfitting.

Secondary endpoints included a continence status at week 1 and at 30 days postoperatively, and erectile function. Exploratory endpoints included continence-related sub-scores of the ICIQ-SF (items Q1–Q3), decision regret, and adherence; exploratory analyses included the multivariable modeling of predictors of continence recovery. Secondary and exploratory endpoints were analyzed using analogous non-parametric or logistic regression models as appropriate. Within-group changes in the continence status over time were assessed using McNemar tests. Between-group comparisons at individual postoperative time points were conducted using logistic regression or non-parametric tests, depending on data distribution.

The effect size for the primary endpoint was expressed using the Phi coefficient (φ) derived from the chi-square test of independence and interpreted according to conventional thresholds for small, moderate, and large effects [27].

For the primary endpoint, a two-sided *p*-value < 0.05 was considered statistically significant. For secondary endpoints, a more stringent significance threshold of *p* < 0.01 was applied to account for multiple testing. All analyses were performed using SPSS version 29.0 (IBM Corp., Armonk, NY, USA). Detailed endpoint definitions, statistical procedures, and additional supporting analyses are provided in the Appendix A.

Missing data were handled using a modified intention-to-treat (mITT) approach, including all randomized patients who underwent surgery and provided at least one postoperative assessment. No formal imputation of missing data was performed. Sensitivity analyses, including best-case and worst-case scenarios, were conducted to assess the robustness of the primary endpoint with respect to the missing data.

## 3. Results

### 3.1. Participant Characteristics and Adherence

**Figure 2:**
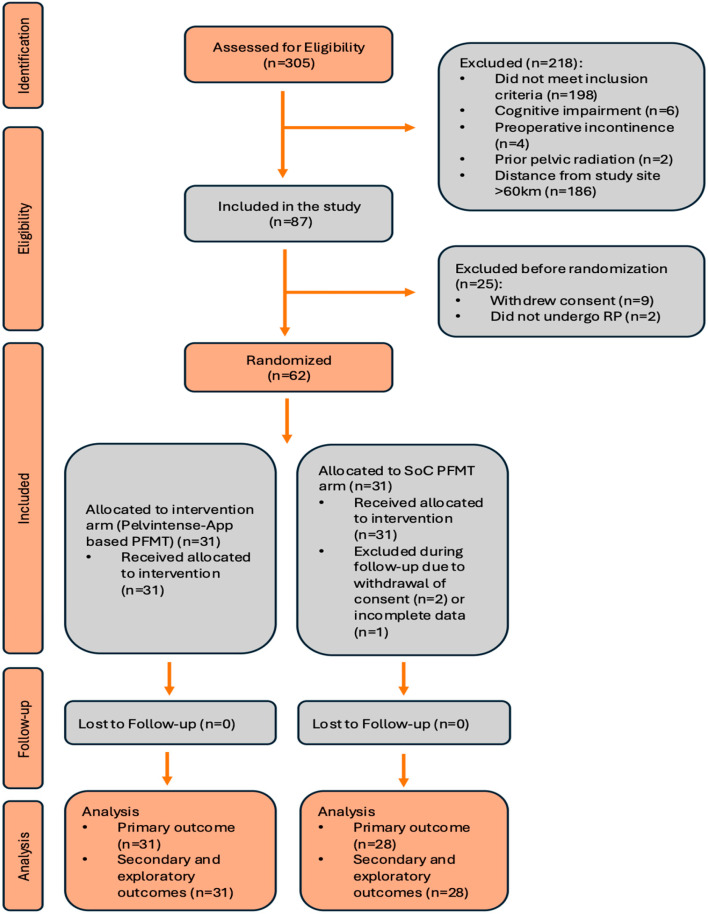
CONSORT flow diagram representing the identification, enrollment, allocation, follow-up, and endpoint analyses of the Pelvintense randomized controlled trial. Patients were screened at three urological centers. PFMT: pelvic floor muscle training, QoL: quality of life, RP: radical prostatectomy, and SUI: stress urinary incontinence.

A total of 62 patients were randomized, with 31 allocated to the app-based perioperative PFMT arm and 31 to the standard physiotherapist-guided arm. Three patients in the control arm withdrew consent for data usage after randomization and were therefore excluded from the evaluable dataset in accordance with ethical requirements. The modified intention-to-treat population comprised 59 patients, including 31 in the intervention arm and 28 in the control arm (Figure 2).

Baseline demographic, oncologic, and surgical characteristics were well balanced between groups (Table 1). The median age was 68 years in the intervention arm and 70 years in the control arm (*p* = 0.284). BMI, PSA, baseline IIEF-5, IPSS-Q8, ASA classification, surgical approach, nerve-sparing status, pathological stage, lymph node yield, and positive surgical margin rates did not differ significantly between groups (all *p* > 0.15). The distribution of surgeon experience was comparable. Postoperative complications were predominantly low grade and did not differ significantly between study arms. The rates of inpatient rehabilitation and adjuvant oncologic therapies were similar between groups.

Presurgical adherence to perioperative PFMT was significantly higher in the intervention arm compared with the control arm (96.8% vs. 67.9%, *p* = 0.003). Post-surgical adherence remained high in both groups without statistically significant differences (100% vs. 96.4%, *p* = 0.289). Additional baseline characteristics, postoperative variables, and supporting analyses are provided in the Appendix A.

### 3.2. Primary Outcome

At 90 days after surgery, continence, defined as ICIQ-SF Q1 = 0, was achieved in 74.2% of patients in the intervention arm compared with 21.4% in the control arm (*p* < 0.001) (Table 2).

The absolute risk reduction was 52.8% (95% CI: 17.2% to 76.1%), corresponding to a relative risk of 3.46 (95% CI: 1.65 to 7.25). The number needed to treat was two (95% CI: 2 to 6), indicating that the treatment of two patients with the app-based perioperative PFMT program resulted in one additional continent patient at 90 days compared with standard care.

In univariable logistic regression, app-based perioperative PFMT was strongly associated with continence recovery (OR 10.54, 95% CI: 3.15 to 35.32; *p* < 0.001). In the multivariable model including all variables associated with the primary endpoint at *p* < 0.20 in univariable analyses, participation in the app-based intervention remained independently associated with continence recovery at 3 months (adjusted OR 13.80, 95% CI: 3.22 to 59.12; *p* < 0.001) (Table 3). The overall model demonstrated adequate fit (Hosmer–Lemeshow *p* = 0.406) and substantial explanatory capacity (Nagelkerke R^2^ = 0.402). Surgical technique and the distribution of unilateral nerve-sparing, bilateral nerve-sparing, and non-nerve-sparing procedures were not significantly associated with the continence outcomes in the multivariable analysis.

To evaluate the robustness of the primary endpoint against missing outcome data in the control arm, a conservative worst-case sensitivity analysis was performed if all three patients who withdrew consent achieved continence at 90 days, which yielded consistent results across different assumptions regarding missing data, supporting the robustness of the primary outcome. Under this assumption, the continence rate in the control arm increased to 29.0% (9/31). The absolute risk reduction remained 45.2% (95% CI: 10.2% to 70.2%), corresponding to a relative risk of 2.56 (95% CI: 1.42 to 4.60) and a number needed to treat of three (95% CI: 2 to 10). The treatment effect, therefore, remained clinically meaningful and statistically robust under this conservative assumption.

In the complementary best-case scenario, assuming all three withdrawn patients were incontinent, the absolute risk reduction was 54.8% (95% CI: 20.5% to 77.1%), with a relative risk of 3.83 (95% CI: 1.81 to 8.10) and a number needed to treat of two (95% CI: 2 to 5). These analyses confirm the stability of the observed treatment effect.

### 3.3. Secondary and Exploratory Outcomes

At one week after the urinary catheter removal, continence was achieved in 25.8% of patients in the intervention arm and 10.7% in the control arm (*p* = 0.124, Table 2). The absolute risk reduction was 15.1% (95% CI: −13.5% to 39.5%), consistent with the absence of a statistically significant between-group difference at this early time point.

At 30 days postoperatively, continence rates were 45.2% in the intervention arm and 3.6% in the control arm (*p* < 0.001, Table 2). The absolute risk reduction was 41.6% (95% CI: 11.4% to 61.6%), corresponding to a relative risk of 12.65 (95% CI: 1.78 to 90.07) and a number needed to treat of three (95% CI: 2 to 9).

**Figure 3:**
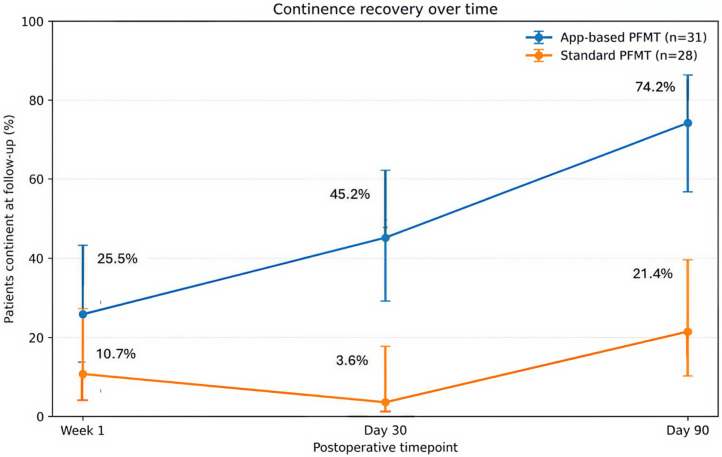
Continence recovery over time in patients participating in the app-based PFMT and standard PFMT.

Within the intervention arm, continence improved significantly from week 1 to day 90 (*p* < 0.001) and from day 30 to day 90 (*p* = 0.022). In contrast, no statistically significant improvement across the postoperative time points was observed in the control arm (Figure 3).

Erectile function, as measured by IIEF-5, did not differ significantly between groups at 30 or 90 days (Table 4).

QoL assessed by IPSS-Q8 was consistently lower in the intervention arm at postoperative time points. At 90 days, the between-group difference reached statistical significance (*p* = 0.004). The difference at 30 days (*p* = 0.017) did not meet the prespecified adjusted threshold for secondary outcomes and is therefore interpreted as exploratory.

Overall decision regret scores at 90 days did not differ significantly between groups. Selected individual items demonstrated lower regret in the intervention arm; however, these findings are exploratory and should be interpreted accordingly. The proportion of patients with clinically relevant decision regret, defined as a total decision regret scale score greater than 15, was 11.1% in the intervention arm and 25.9% in the control arm (*p* = 0.16). Complete secondary and exploratory outcome data are presented in Table 2 and Table 4.

## 4. Discussion

In the era of digital medicine, mobile health applications are increasingly integrated into perioperative care to enhance clinical outcomes and patient engagement. Digital medicine technologies have demonstrated measurable benefits across various areas of healthcare, including chronic disease management and behavioral interventions such as smoking cessation and weight control [28].

The randomized controlled trial Pelvintense indicates that a modular, app-based perioperative PFMT program is associated with a significant acceleration of continence recovery, an improvement in QoL, and enhanced training adherence after RP compared with conventional physiotherapist-guided training. These findings provide the first evidence from a multi-center randomized controlled trial that a structured digital approach incorporating both prehabilitation and postoperative training can meaningfully enhance early functional recovery after surgery.

Beyond statistical significance, the observed treatment effect was clinically substantial. At 90 days, continence was achieved in nearly three-quarters of the patients assigned to the app-based intervention compared with just over one-fifth in the control group, translating into an absolute risk reduction exceeding 50% and a number needed to treat of two. Sensitivity analyses applying conservative worst-case and best-case assumptions for missing outcome data in the control arm confirmed the robustness of this finding, indicating that the observed benefit is unlikely to be driven by attrition or analytic assumptions. In multivariable analysis, adjusting for relevant clinical and surgical factors, app-based PFMT remained the dominant independent predictor of early continence recovery, underscoring the strength and consistency of the intervention effect. Continence recovery following RP is influenced by multiple anatomical and surgical factors, including urethral length, nerve-sparing techniques, and reconstructive approaches. In this context, the concept of the “pentafecta”—typically encompassing cancer control, urinary continence, erectile function, absence of complications, and negative surgical margins—provides a framework for evaluating postoperative outcomes and highlights that continence recovery represents only one component influenced by both surgical and rehabilitative factors. Within this framework, the observed differences in continence recovery in our study are unlikely to be explained by surgical factors but rather suggest a strong contribution of structured perioperative rehabilitation and an optimized implementation of pelvic floor muscle training.

Our results confirm the clinical relevance of perioperative PFMT and align with the previous data on its efficacy when initiated prior to surgery and continued thereafter. Prior meta-analyses indicate that combined preoperative and postoperative PFMT accelerates continence recovery compared with postoperative-only regimens [29]. A systematic review by Brea-Gómez et al. further underscores the importance of early functional activation, demonstrating lower incontinence rates in patients receiving preoperative biofeedback-based training, with odds ratios of 0.51 at three months, 0.40 at three to six months, and 0.29 at six to twelve months after surgery compared with the standard of care [20]. The Pelvintense program operationalizes these principles through digital delivery, interactive intensity-progressive modules, and structured feedback, achieving a continence rate of 74% at 90 days compared with 21% in the standard-of-care group. The magnitude of the observed effect is consistent with, and extends beyond, prior analog interventions.

The observed differences in continence recovery between patients using the Pelvintense app and patients conducting standard-of-care physical therapy may be explained by several established physiological and behavioral mechanisms underlying pelvic floor rehabilitation. PFMT relies on neuromuscular adaptation, motor learning, and early reactivation of the pelvic floor following surgical trauma. Structured and repeated activation of the pelvic floor musculature may enhance neuromuscular coordination and improve sphincter function. In addition, digital delivery may facilitate more consistent training, reinforce correct technique, and promote behavioral adherence, all of which are critical determinants of PFMT efficacy. Structured progression to higher intensity and more complex pelvic floor exercises, including contraction, relaxation, and coordination, tailored to individual progress within the app-based program, may further enhance the overall efficacy of PFMT. Together, these mechanisms provide the clinical rationale for a structured, perioperative PFMT approach and support the concept that both timing and implementation quality are critical determinants of functional recovery.

Notably, the current evidence base for digital pelvic floor muscle training in men remains limited, with most available studies focusing on conventional physiotherapy approaches. High-quality randomized data evaluating fully digital, app-based interventions for male urinary incontinence are not available yet, underscoring the relevance of the present study. While comparative data addressing app-based PFMT are lacking, the outcomes achieved in the Pelvintense study are consistent with the emerging evidence from smaller digital interventions, suggesting that app-based platforms may effectively support behavioral changes as well as improve adherence and engagement in various disease settings, such as obesity or mental health disorders [29,30]. Importantly, the present study demonstrates that such benefits can be achieved across multiple centers using a standardized digital platform, highlighting the feasibility of integrating app-based rehabilitation into routine urologic care pathways rather than restricting it to highly specialized settings.

In parallel, a recent multi-center study systematically evaluated the perceived importance of various supportive measures offered to patients undergoing RP [31]. Of the fifteen interventions assessed, six were rated as highly relevant, with preoperative and postoperative instruction in PFMT emerging as the most frequently endorsed. Notably, both were identified by patients as the most important components of their perioperative care. These findings underline a clear patient-driven mandate to optimize and prioritize the structured delivery of perioperative PFMT throughout the surgical pathway.

Compared with traditional physical therapy, the Pelvintense application offers a self-directed, modular training concept that can be flexibly adapted to individual progress. Core features such as motivational reinforcement, exercise reminders, and audiovisual guidance likely contributed to the markedly higher preoperative adherence observed in the intervention group and to the sustained engagement during the postoperative phase. The ability to train independently may help overcome structural limitations, such as restricted access to physiotherapy services, while maintaining or improving clinical outcomes.

The temporal pattern of recovery observed in this trial is also clinically informative. While differences in continence rates at one week after the catheter removal did not reach statistical significance, a pronounced separation between groups was evident by 30 days, with a low number needed to treat. This suggests that digital perioperative PFMT primarily accelerates functional recovery rather than merely shifting long-term continence rates, a finding that is biologically plausible given the neuromuscular adaptation required for pelvic floor reconditioning.

Beyond continence, patients in the intervention group reported better continence-related QoL and lower decision regret in selected domains. Although these secondary and exploratory outcomes should be interpreted cautiously, they suggest that improved functional recovery may translate into broader patient-centered benefits, including greater perceived control and satisfaction during the postoperative course. As expected, at a follow-up of 90 days, erectile function did not differ between groups. The absence of differences in erectile function between groups may reflect the relatively short follow-up period, as recovery of erectile function following RP is known to occur over a longer timeframe and is influenced by additional factors, such as nerve-sparing techniques. In addition, PFMT is not primarily designed to directly influence neurovascular recovery, which is a key determinant of erectile function, further limiting the likelihood of detecting early differences between groups. Accordingly, the absence of a detectable effect on erectile function at this early time point is not unexpected. Longer-term follow-up will be required to assess the potential effects on sexual recovery.

Several strengths of this trial merit emphasis. The study was conducted as a prospectively randomized, multi-center trial in a pragmatic surgical setting, including patients treated with different operative approaches and by surgeons with varying levels of experience. The intervention was standardized and reproducible, and effect estimates were reported using absolute and relative measures that facilitate clinical interpretation. The use of sensitivity analyses to address missing outcome data further enhances transparency and methodological robustness. In addition, the inclusion of patient-relevant outcomes such as QoL and decision regret provides a multidimensional perspective on treatment impact beyond the traditional functional endpoints.

At the same time, several limitations should be acknowledged. First, the relatively small sample size limits the ability to perform detailed subgroup analyses and to draw firm conclusions regarding secondary outcomes. Second, the unblinded nature of the intervention and reliance on patient-reported continence outcomes may introduce potential sources of bias. As is typical for behavioral and rehabilitative interventions, performance bias cannot be excluded, and differences in motivation, engagement, and adherence between groups may have contributed to the observed effect size. However, these factors should not be viewed primarily as sources of bias, but rather as core components of the intervention itself. The app-based PFMT program was specifically designed to enhance engagement, support adherence, and provide structured, progressive training through modular content and standardized exercises. Therefore, the observed effect reflects not only the mode of delivery but the integrated intervention strategy, including behavioral activation, training structure, and execution of exercises, which may differ from conventional physiotherapy-guided approaches. Third, attrition and incomplete patient-reported outcome data represent another potential source of bias. However, completion rates were high, and the sensitivity analyses suggested that missing data did not materially affect the primary findings. Fourth, the relatively low continence rate observed in the control group compared with some reports in the literature may be explained, at least in part, by the strict definition of continence used in this study (ICIQ-SF Question 1 = 0), which represents a more stringent criterion than pad-based definitions commonly applied elsewhere. Importantly, this definition was applied consistently across both groups and therefore does not affect the internal comparison between study arms. Fifth, the requirement for digital literacy may limit generalizability to certain patient populations. In addition, while adherence was high overall, only approximately half of the participants completed all progressive app modules, indicating that sustained engagement remains a challenge for digital interventions. Finally, the relatively short follow-up period of 90 days post RP, focusing on early recovery, limits conclusions regarding the long-term continence outcomes. While previous studies suggest that early benefits of perioperative PFMT may persist over time [19], confirmation in studies with extended follow-up is warranted. Generalizability may also be influenced by the selected study population, including patients who were continent preoperatively and treated at specialized centers [32].

From a broader perspective, these findings have implications for the evolving role of AI in urologic oncology. While the Pelvintense application is not an AI-based system in its current form, it represents a structured, data-generating digital platform that captures longitudinal information on patient behavior, adherence, and functional recovery. Such datasets are essential for the development of AI-driven models capable of predicting continence recovery, identifying patients at risk for delayed functional outcomes, and dynamically adapting rehabilitation protocols.

In this context, future integration of machine learning approaches could enable personalized training intensity, timing, and progression based on individual recovery trajectories and baseline risk profiles. Moreover, combining digital rehabilitation data with clinical, surgical, and potentially image-derived variables may facilitate multimodal predictive models that align with emerging concepts of precision medicine in urologic oncology. This paradigm shift—from standardized rehabilitation to data-driven, adaptive recovery pathways—may represent an important extension of AI applications beyond diagnostics and prognostics toward functional outcome optimization.

Importantly, such approaches would not replace established perioperative care but rather augment clinical decision-making by providing real-time, patient-specific insights. The present study not only shows the clinical efficacy of digital intervention but also establishes a scalable infrastructure for future AI-enabled rehabilitation strategies in cancer care.

Future studies should validate these findings in larger cohorts, extend follow-up to assess the durability of the benefits, and evaluate cost-effectiveness and health–economic impact. Identifying patient subgroups most likely to benefit from digital rehabilitation and integrating such programs into comprehensive perioperative care ecosystems may further enhance outcomes. Taken together, this study provides robust early evidence that structured, app-based perioperative PFMT can meaningfully improve early continence recovery after RP and supports its consideration as a scalable adjunct to standard care.

## 5. Conclusions

This multicenter randomized controlled trial suggests that a structured, modular app-based perioperative PFMT program is associated with improved early continence recovery following RP, as well as improved continence-related QoL, compared with standard physiotherapy-guided care. The observed effect was clinically meaningful and consistent across sensitivity analyses, supporting the robustness of the findings. The treatment effect is likely multifactorial and may be driven by the training itself (including modular and individual progression of contraction, relaxation, and combination exercises) in addition to structured guidance, higher adherence, and continuous patient engagement. App-based perioperative prehabilitation and rehabilitation may represent a scalable and resource-efficient approach to translating guideline-recommended supportive care into routine practice and could complement contemporary digitally enabled urologic oncology pathways. Further studies are warranted to confirm long-term durability, cost-effectiveness, and optimal patient selection, as well as to better define the role of digital PFMT interventions within multimodal postoperative rehabilitation strategies. In addition, the structured digital nature of the intervention provides a foundation for the future integration of artificial intelligence, based approaches aimed at personalized rehabilitation and prediction of functional recovery in urologic oncology.

## Figures and Tables

**Table 1:** Descriptive summary of the baseline demographics, clinical and pathological characteristics, and training of patients in the app-based PFMT and standard PFMT groups.

Parameter	Entire Cohort (*n* = 59)	App-Based PFMT (*n* = 31)	Standard PFMT (*n* = 28)	*p*
Age (years, median [IQR])	70 (65–73)	68 (62–73)	70 (67–74.5)	0.284
BMI (kg/m^2^, median [IQR])	26.2 (24.7–28.7)	25.2 (23.5–29.3)	27 (25.4–28.1)	0.227
PSA (ng/mL, median [IQR])	8.4 (5.8–10.4)	8.6 (6.6–10.2)	7.8 (5.7–10.8)	0.693
IIEF-5 sum score (randomization) (median [IQR])	15 (7–20)	15 (10–19)	11 (5–20)	0.149
QoL, IPSS-Q8 (median [IQR])	1 (1–2)	1 (1–2)	1 (1–3)	0.169
ASA score ASA 1 ASA 2 ASA 3	1 (1.7%) 47 (79.7%) 11 (18.6%)	0 25 (80.6%) 6 (19.4%)	1 (3.6%) 22 (78.6%) 5 (17.9%)	0.568
Preoperative TURP (*n*, %)	5 (8.5%)	2 (6.5%)	3 (10.7%)	0.557
Type of RP (*n*, %) RALP Laparoscopic Open	43 (72.9%) 13 (22%) 3 (5.1%)	23 (74.2%) 6 (19.4%) 2 (6.5%)	20 (71.4%) 7 (25%) 1 (3.6%)	0.791
Lifetime expertise of the Surgeon (*n*, %) ≤50 RPs 51–100 RPs >100 RPs	11 (18.6%) 17 (28.8%) 31 (52.5%)	7 (22.6%) 9 (29%) 15 (48.4%)	4 (14.3%) 8 (28.6%) 16 (57.1%)	0.684
Nerve-sparing (*n*, %) No Unilateral Bilateral	3 (5.1%) 10 (16.9%) 46 (78%)	1 (3.2%) 7 (22.6%) 23 (74.2%)	2 (7.1%) 3 (10.7%) 23 (82.1%)	0.410
Lymph node count (median [IQR])	8 (6–13)	9 (7–13)	8 (6–13)	0.692
pT stage (*n*, %) pT2a pT2c pT3a pT3b	3 (5.1%) 33 (55.9%) 15 (25.4%) 8 (13.6%)	3 (9.7%) 18 (58.1%) 6 (19.4%) 4 (12.9%)	0 15 (53.6%) 9 (32.1%) 4 (14.3%)	0.292
pN1 stage (*n*, %)	2 (3.4%)	1 (3.2%)	1 (3.6%)	0.942
Positive Surgical Margins (*n*, %)	17 (28.8%)	10 (32.3%)	7 (25%)	0.539
ISUP Gleason group (*n*, %) 2 3 4 5	37 (62.7%) 15 (25.4%) 3 (5.1%) 4 (6.8%)	21 (67.7%) 5 (16.1%) 3 (9.7%) 2 (6.5%)	16 (57.1%) 10 (35.7%) 0 2 (7.1%)	0.158
Anastomosis insufficiency on first cystogram (*n*, %)	3 (5.1%)	1 (3.2%)	2 (7.1%)	0.494
Inpatient rehabilitation (*n*, %)	28 (47.5%)	14 (45.2%)	14 (50%)	0.710
Adherence to the presurgical PFMT (*n*, %)	49 (83.1%)	30 (96.8%)	19 (67.9%)	0.003
Adherence to the post-surgical PFMT (*n*, %)	58 (98.3%)	31 (100%)	27 (96.4%)	0.289

ASA: American Society of Anesthesiologists; BMI: body mass index; IPSS-Q8: International Prostate Symptom Score, question 8; IQR: interquartile range; ISUP: International Society of Urological Pathology; PFMT: pelvic floor muscle training; PSA: prostate-specific antigen; QoL: quality of life; RALP: robot-assisted laparoscopic prostatectomy; RP: radical prostatectomy; TURP: transurethral resection of the prostate.

**Table 2:** Comparison of the continence outcomes between the app-based PFMT and standard PFMT groups at key postoperative time points (week 1, day 30, and day 90 after urinary catheter removal). Outcomes are reported as medians with interquartile ranges (IQR) and percentages for categorical variables. The statistical significance was determined using the Mann–Whitney U-test for ordinal outcomes and the chi-square test for categorical data.

Outcome	App-Based PFMT (*n* = 31)	Standard PFMT (*n* = 28)	*p*
Week 1 after urinary catheter removal			
Median ICIQ-SF, Q1 [IQR])	1 (0–2)	2 (1.25–3.75)	0.024
Median ICIQ-SF, Q2 [IQR])	2 (2–2)	2 (2–4)	0.090
Median ICIQ-SF, Q3 [IQR])	2 (0–4)	4 (2–6.75)	0.035
ICIQ-SF score, Q1–Q3 [IQR])	6 (3–9)	8 (6.25–13)	0.017
Continence (ICIQ-SF Q1 = 0) (*n*, %)	8 (25.8%)	3 (10.7%)	0.124
Day 30 post-surgically			
Median ICIQ-SF, Q1 [IQR])	1 (0–1)	2 (1–3)	<0.001
Median ICIQ-SF, Q2 [IQR])	2 (0–2)	2 (2–2)	<0.001
Median ICIQ-SF, Q3 [IQR])	1 (0–2)	4 (2.25–6)	<0.001
ICIQ-SF score, Q1–Q3 [IQR])	4 (0–6)	8 (5.25–12)	<0.001
Continence (ICIQ-SF Q1 = 0) (*n*, %)	14 (45.2%)	1 (3.6%)	<0.001
Day 90 post-surgically			
Median ICIQ-SF, Q1 [IQR])	0 (0–1)	1 (1–3.75)	<0.001
Median ICIQ-SF, Q2 [IQR])	0 (0–2)	2 (2–2)	<0.001
Median ICIQ-SF, Q3 [IQR])	0 (0–1)	3 (1–4)	<0.001
ICIQ-SF score, Q1–Q3 [IQR])	1 (0–4)	6.5 (4–9.75)	<0.001
Continence (ICIQ-SF Q1 = 0) (*n*, %)	23 (74.2%)	6 (21.4%)	<0.001

d30: day 30 postoperatively; d90: day 90 postoperatively; ICIQ-SF: International Quality of Life Questionnaire, short form; IQR: interquartile range; PFMT: pelvic floor muscle training.

**Table 3:** Univariable and multivariable binary logistic regression analyses identifying predictors of continence at 90 days postoperatively, defined as ICIQ-SF Q1 = 0. Results are reported as odds ratios (OR) with corresponding 95% confidence intervals (CI) and *p*-values. All variables demonstrating an association with the primary endpoint at *p* < 0.20 in univariable analyses were entered into the multivariable model. No automated variable selection procedures were applied.

Criterion	Univariate Model (OR [95% CI], *p*)	Multivariable Model (OR [95% CI], *p*)
Age (years), cont.	OR 0.98 (95% CI: 0.91–1.07), *p* = 0.722	-
BMI (kg/m^2^), cont.	OR 0.97 (95% CI: 0.85–1.12), *p* = 0.715	-
Preoperative PSA (ng/mL), cont.	OR 1.02 (95% CI: 0.90–1.16), *p* = 0.770	-
ASA score 3 (vs. 1–2)	OR 1.30 (95% CI: 0.35–4.86), *p* = 0.692	-
Preoperative TURP (vs. no TURP)	OR 0.67 (95% CI: 0.10–4.31), *p* = 0.670	-
RALP (vs. other types of RP)	OR 0.48 (95% CI: 0.15–1.54), *p* = 0.215	-
Surgeon’s expertise > 100 RPs (vs. others)	OR 0.41 (95% CI: 0.14–1.17), *p* = 0.094	OR 0.42 (95% CI: 0.10–1.76), *p* = 0.236
Bilateral nerve-sparing (vs. others)	OR 0.34 (95% CI: 0.09–1.27), *p* = 0.109	OR 0.46 (95% CI: 0.09–2.41), *p* = 0.356
pT3 stage (vs. pT2)	OR 0.92 (95% CI: 0.32–2.61), *p* = 0.871	-
pN1 stage (vs. pN0)	OR 1.04 (95% CI: 0.06–17.38), *p* = 0.981	-
Positive surgical margins (vs. negative)	OR 1.73 (95% CI: 0.55–5.41), *p* = 0.347	-
ISUP Gleason group 3–5 (vs. ISUP Gleason group 2)	OR 0.79 (95% CI: 0.27–2.27), *p* = 0.662	-
Anastomotic insufficiency (vs. no)	OR 0.00 (95% CI: 0.00–0.00), *p* = 0.999	-
Inpatient rehabilitation (vs. others)	OR 0.62 (95% CI: 0.22–1.73), *p* = 0.359	-
Presurgical PFMT (vs. no)	OR 2.64 (95% CI: 0.61–11.41), *p* = 0.194	OR 0.49 (95% CI: 0.07–3.53), *p* = 0.477
App-based PFMT (vs. standard PFMT)	OR 10.54 (95% CI: 3.15–35.32), *p* < 0.001	OR 13.80 (95% CI: 3.22–59.12), *p* < 0.001

95% CI: 95% confidence interval, ASA: American Society of Anesthesiologists, BMI: body mass index, OR: odds ratio, PFMT: pelvic floor muscle training, PSA: prostate-specific antigen, RALP: robot-assisted laparoscopic prostatectomy, RP: radical prostatectomy, TURP: transurethral resection of the prostate.

**Table 4:** Quality of life (assessed via IPSS-Q8) at w1, d30, and d90, erectile function (assessed via IIEF-5 scores) at 30 and 90 days postoperatively, and decision regret (assessed via decision regret scores) at 90 days postoperatively in the app-based PFMT and standard PFMT groups. The data are expressed as medians with interquartile ranges (IQR). Statistical analyses were performed using the Mann–Whitney U-test for ordinal outcomes.

Outcome	App-Based PFMT (*n* = 31)	Standard PFMT (*n* = 28)	*p*
Quality of life			
QoL, IPSS-Q8, w1 (median [IQR])	2 (0.75–4.25)	3 (2–5)	0.056
QoL, IPSS-Q8, d30 (median [IQR])	2 (0–3)	3 (2–4.75)	0.017
QoL, IPSS-Q8, d90 (median [IQR])	1 (0–2)	2 (1.25–3)	0.004
Erectile function			
IIEF-5 score, d30 (median [IQR])	5 (5–10)	5 (5–14)	0.679
IIEF-5 score, d90 (median [IQR])	5 (5–12)	5 (5–8)	0.815
Decision regret			
Decision regret score sum score (median [IQR])	5 (0–10)	10 (0–20)	0.077
Decision regret score Q1 (median [IQR])	1 (1–1)	1 (1–2)	0.021
Decision regret score Q2 inverse (median [IQR])	1 (1–1)	1 (1–2)	0.551
Decision regret score Q3 (median [IQR])	1 (1–1)	1 (1–2)	0.272
Decision regret score Q4 inverse (median [IQR])	1 (1–2)	2 (1–3)	0.024
Decision regret score Q5 (median [IQR])	1 (1–1)	1 (1–1)	0.693

d30: day 30 postoperatively; d90: day 90 postoperatively; IIEF-5: International Index of Erectile Function-5 items; IPSS-Q8: International Prostate Symptom Score, question 8; IQR: interquartile range; PFMT: pelvic floor muscle training; QoL: quality of life.

## Data Availability

The data supporting the findings of this study are presented within the article and its Appendix A. Due to data protection regulations and ongoing regulatory evaluation processes (including DiGA submission to the German Federal Institute for Drugs and Medical Devices, BfArM), the full dataset is not publicly available. Additional data may be made available from the corresponding author upon reasonable request and subject to applicable ethical, legal, and regulatory requirements.

## Abbreviations

The following abbreviations are used in this manuscript:

ASA	American Society of Anesthesiologists
AUA	American Urological Association
BMI	Body Mass Index
CI	Confidence Interval
DRKS	German Clinical Trials Register
EAU	European Association of Urology
ICIQ-SF	International Consultation on Incontinence Questionnaire Short Form
IIEF-5	International Index of Erectile Function–5 items
IPSS	International Prostate Symptom Score
IPSS-Q8	International Prostate Symptom Score, Quality of Life Question
IQR	Interquartile Range
ISUP	International Society of Urological Pathology
mITT	Modified Intention-to-Treat
OR	Odds Ratio
PCa	Prostate Cancer
PFMT	Pelvic Floor Muscle Training
QoL	Quality of Life
RALP	Robot-Assisted Laparoscopic Prostatectomy
RP	Radical Prostatectomy
SUI	Stress Urinary Incontinence
TURP	Transurethral Resection of the Prostate

## Appendix A

**Table A1:** The statistical methods applied in the Pelvintense study.

Endpoints	Hypothesis	Outcome Measure	Statistical Methods
Primary endpoint Continence at 90 days postoperatively, defined as ICIQ-SF Q1 = 0.	The intervention improves continence at 90 days compared with standard physiotherapist-guided training.	Binary definition: ICIQ-SF Q1 = 0 vs. ≥1	Continuous variables are displayed as medians with interquartile ranges (IQRs). Mann–Whitney U tests were used for non-normally distributed continuous variables, and chi-square tests were used for categorical variables. Multivariable logistic regression including all variables associated with the primary endpoint at *p* < 0.20 in univariable analyses; no automated variable selection procedures were applied.
Secondary outcomes at one week, 30 days, and 90 days Continence rates QoL (IPSS-Q8) Decision regret Erectile function	The intervention is associated with improved continence-related quality of life, decision regret, and erectile function compared with standard care at prespecified postoperative time points.	Binary definition: ICIQ-SF Q1 = 0 vs. ≥1 QoL (IPSS-Q8) decision regret scale IIEF-5	McNemar test Mann–Whitney U-test for ordinal outcomes
Adherence at 90 days		Adherence at 90 days, defined as completion of >95% of prescribed training sessions within the preceding 30 days (binary).	Chi-square test

Note: A *p*-value of <0.05 was considered for statistical significance for primary outcomes, while a threshold of <0.01 was applied for secondary outcomes to account for multiple testing.

**Table A2:** Completeness of patient-reported outcomes (ICIQ-SF) from baseline to 90 days.

Data Available for ICIQ-SF	Study Arm			
	Intervention Arm (Pelvintense App) (*n* = 31)		Standard-of-Care Arm (*n* = 28)	
	*n*	(%)	*n*	(%)
Baseline	31	(100)	31	(100)
1 week	31	(100)	28	(87)
30 days	31	(100)	28	(87)
90 days	31	(100)	28	(87)

Note: Patient-reported outcome booklets were given to participants at their hospital visits or were posted 30 and 90 days post-surgery. In some cases of the PRO booklet completion, individual items may not have been reported. Three patients in the control group (standard of care) withdrew their consent for data analysis.

**Table A3:** Completeness of patient-reported outcomes (IPSS-Q8) from baseline to 90 days.

Data Available for IPSS-Q8	Study Arm			
	Intervention Arm (Pelvintense App) (*n* = 31)		Standard-of-Care Arm (*n* = 28)	
	*n*	(%)	*n*	(%)
Baseline	31	(100)	31	(100)
1 week	31	(100)	28	(90.3)
30 days	31	(100)	28	(90.3)
90 days	26	(83.9)	28	(90.3)

Note: Patient-reported outcome booklets were given to participants at their hospital visits or were posted 30 and 90 days post-surgery. In some cases of the PRO booklet completion, individual items may not have been reported. Three patients in the control group (standard of care) withdrew their consent for data analysis.

**Table A4:** Completeness of patient-reported outcomes (decision regret) from week 1, 30 days, and 90 days post-surgery.

Data Available for Decision Regret Scale	Study Arm			
	Intervention Arm (Pelvintense App) (*n* = 31)		Standard-of-Care Arm (*n* = 28)	
	*n*	(%)	*n*	(%)
Baseline	0		0	
1 week	31	(100)	28	(90.3)
30 days	30	(96.8)	27	(87.1)
90 days	27	(87.1)	27	(87.1)

Note: Patient-reported outcome booklets were given to participants at their hospital visits or were posted 30 and 90 days post-surgery. In some cases of the PRO booklet completion, individual items may not have been reported. Three patients in the control group (standard of care) withdrew their consent for data analysis.

**Table A5:** Post-surgical treatments and complication rates (Clavien–Dindo classification).

Parameter	Intervention Arm (Pelvintense App) (*n* = 31)	Standard-of-Care Arm (*n* = 28)
Clavien–Dindo classification, *n* (%) Grade 0 Grade 1 Grade 2 Grade 3a Grade 3b	17 (54.8) 12 (38.7) 1 (3.2) 0 (0) 1 (3.2)	17 (60.7) 7 (25) 4 (14.3) 0 (0) 0 (0)
Adjuvant radiotherapy, *n* (%)	1 (3.2)	0 (0)
Adjuvant hormonal therapy, *n* (%)	2 (6.5)	2 (7.1)

Note: For inferential comparison, Clavien–Dindo grades were dichotomized into Grade 0–1 versus Grade ≥ 2 to provide a clinically meaningful distinction between no or minor complications and at least moderate postoperative morbidity. Using a Pearson chi-square test, no statistically significant difference in complication severity distribution was observed between groups (*p* = 0.320) [Fisher exact (2 × 2): *p* = 0.409].

**Table A6:** Sensitivity analyses for the primary endpoint at 90 days.

Analysis Set	mITT (31 vs. 28)	Worst-Case Scenario *	Best-Case Scenario ^†^
Continence rate intervention	74.2% (23/31)	74.2% (23/31)	74.2% (23/31)
Continence rate control	21.4% (6/28)	29.0% (9/31)	19.4% (6/31)
Absolute risk reduction (95% CI)	52.8% (17.2% to 76.1%)	45.2% (10.2% to 70.2%)	54.8% (20.5% to 77.1%)
Relative risk (95% CI)	3.46 (1.65 to 7.25)	2.56 (1.42 to 4.60)	3.83 (1.81 to 8.10)
Number needed to treat (95% CI)	2 (2 to 6)	3 (2 to 10)	2 (2 to 5)

Note: * the worst-case scenario assumes that all three control-arm patients who withdrew consent achieved continence (ICIQ-SF Q1 = 0) at 90 days. ^†^ the best-case scenario assumes that all three control-arm patients who withdrew consent remained incontinent at 90 days. All effect estimates are based on unadjusted group comparisons.
